# Recursive SVM biomarker selection for early detection of breast cancer in peripheral blood

**DOI:** 10.1186/1755-8794-6-S1-S4

**Published:** 2013-01-23

**Authors:** Fan Zhang, Howard L Kaufman, Youping Deng, Renee Drabier

**Affiliations:** 1 Department of Academic and Institutional Resources and Technology, University of North Texas Health Science Center, Fort Worth, TX, USA; 2 Department of Forensic and Investigative Genetics, University of North Texas Health Science Center, Fort Worth, TX, USA; 3 Rush University Cancer Center, Rush University Medical Center, Chicago, IL 60612, USA; 4 Department of General Surgery and Immunology and Microbiology, Rush University Medical Center, Chicago, IL 60612, USA; 5 Department of Internal Medicine and Biochemistry, Rush University Medical Center, Chicago, IL 60612, USA

## Abstract

**Background:**

Breast cancer is worldwide the second most common type of cancer after lung cancer. Traditional mammography and Tissue Microarray has been studied for early cancer detection and cancer prediction. However, there is a need for more reliable diagnostic tools for early detection of breast cancer. This can be a challenge due to a number of factors and logistics. First, obtaining tissue biopsies can be difficult. Second, mammography may not detect small tumors, and is often unsatisfactory for younger women who typically have dense breast tissue. Lastly, breast cancer is not a single homogeneous disease but consists of multiple disease states, each arising from a distinct molecular mechanism and having a distinct clinical progression path which makes the disease difficult to detect and predict in early stages.

**Results:**

In the paper, we present a Support Vector Machine based on Recursive Feature Elimination and Cross Validation (SVM-RFE-CV) algorithm for early detection of breast cancer in peripheral blood and show how to use SVM-RFE-CV to model the classification and prediction problem of early detection of breast cancer in peripheral blood.

The training set which consists of 32 health and 33 cancer samples and the testing set consisting of 31 health and 34 cancer samples were randomly separated from a dataset of peripheral blood of breast cancer that is downloaded from Gene Express Omnibus. First, we identified the 42 differentially expressed biomarkers between "normal" and "cancer". Then, with the SVM-RFE-CV we extracted 15 biomarkers that yield zero cross validation score. Lastly, we compared the classification and prediction performance of SVM-RFE-CV with that of SVM and SVM Recursive Feature Elimination (SVM-RFE).

**Conclusions:**

We found that 1) the SVM-RFE-CV is suitable for analyzing noisy high-throughput microarray data, 2) it outperforms SVM-RFE in the robustness to noise and in the ability to recover informative features, and 3) it can improve the prediction performance (Area Under Curve) in the testing data set from 0.5826 to 0.7879. Further pathway analysis showed that the biomarkers are associated with Signaling, Hemostasis, Hormones, and Immune System, which are consistent with previous findings. Our prediction model can serve as a general model for biomarker discovery in early detection of other cancers. In the future, Polymerase Chain Reaction (PCR) is planned for validation of the ability of these potential biomarkers for early detection of breast cancer.

## Background

Breast cancer is the most common type of cancer among women in the United States [1]. Early detection is key to the successful treatment of breast cancer. Traditional methods most used for early detection have been regular and periodic self examination and annual or biannual check-ups using mammography and analysis of tissue biopsies. However, early cancer detection and treatment are still challenging. One reason is that mammography as a screening tool for early detection has many drawbacks. For example, mammography may not detect small tumors, and is often unsatisfactory for younger women, who typically have dense breast tissue. Another reason is that obtaining tissue biopsies can be difficult for reasons including small size of lump, lack of available medical facilities, and patients' reluctance to undergo invasive procedures due to potential scaring and financial costs. Moreover, the fact that breast cancer is not a single homogeneous disease but consists of multiple disease states, each arising from a distinct molecular mechanism and having a distinct clinical progression path [2], makes the disease difficult to detect in early stages.

To address these issues, a novel and minimally invasive test that uses easily obtained peripheral blood for breast cancer detection has been developed [3,4]. For example, Sharma *et al. *used microarrays and nearest-shrunken-centroid method to analyze the expression pattern of 1,368 genes in peripheral blood cells of 24 women with breast cancer and 32 women with no sign of this disease. The study found that a blood-based gene expression test can be developed to detect breast cancer early in asymptomatic patients [4]. Aaroe *et al. *collected peripheral blood from 67 breast cancer samples and 63 normal samples, identified a set of 738 differentially expressed probes, and achieved an estimated prediction accuracy of 79.5% with a sensitivity of 80.6% and a specificity of 78.3% [3].

There is a need for more reliable diagnostic tools for early detection of breast cancer in peripheral blood which can achieve high prediction accuracy with as few genes as possible, and to reduce the required examination of a large number of genes which increases the dimensionality, computational complexity, and clinical cost of diagnosis [5].

Support Vector Machine Recursive Feature Elimination (SVM-RFE) approach for gene selection proposed by Guyon [6] is one of the most effective feature selection methods which has been successfully used in selecting informative genes for cancer classification. It is a backward selection approach that selects genes according to their influence (weight) on a support vector machine. First it calculates ranking criteria based on the SVM weights. Then it eliminates features with the smallest ranking criterion. Lastly it repeats the process until a highest classification accuracy is achieved.

SVM-RFE is used to find discriminate relationships within clinical datasets and within gene expression datasets created from micro-arrays of tumor versus normal tissues. However, the feature elimination method is sensitive to small perturbations of the training set. The features it extracts from training set might not have good prediction performance in an independent testing set. This is probably caused by overfitting which arises when 1) the number of features is large and the number of training patterns is comparatively small or 2) some regularities appear in the training data that do not appear in the test data. In order to avoid the overfitting and gain best prediction accuracy for the testing set, we built an SVM based on Recursive Feature Elimination and Cross Validation (SVM-RFE-CV) to extract optimal features.

We propose for the first time a multi-marker panel development solution for early detection of breast cancer in peripheral blood by using a SVM-RFE-CV, and show how to use SVM-RFE-CV to model the classification and prediction problem of early detection of breast cancer in peripheral blood.

We compared the classification and prediction performance of SVM-RFE-CV with that of SVM and SVM Recursive Feature Elimination (SVM-RFE) and found that 1) the SVM-RFE-CV is suitable for analyzing noisy high-throughput microarray data, 2) it outperforms SVM-RFE in the robustness to noise and in the ability to recover informative features, and 3) it can improve the prediction performance (Area Under Curve) in the testing data set from 0.5826 to 0.7879.

## Materials and methods

### Peripheral blood data collection

We downloaded the 130 samples of peripheral blood data through the GEO database, which are publically available with the accession number GSE16443 [3] and were collected to originally determine the potential of gene expression profiling of peripheral blood cells for early detection of blood cancer. It consists of 130 samples with 67 cases and 63 controls. We randomly divided the 130 samples into two groups: group A as a training set and group B as a testing set (Table 1).

**Table 1:** statistics of samples

	#health	#cancer	#total
Training set	32	33	65
Testing set	31	34	65
Total	63	67	130

### Normalization

Normalization per sample was used to normalize the data. First, log ratio base 2 transformation was used to transform the data. And then for each probe the median of the log summarized values from all the samples were calculated and subtracted from each of the samples.

### Linear mixed model

We used the ABI Human Genome Survey Microarray Version 2 to manage and map probe IDs. A full factorial model was used to represent the fixed effect and the random effect which are used to account for group and probe. The expression log ratios value is the final quantity that is fit by a separate analysis of the variance (ANOVA) statistical model for each probe as *y_il _*using the following:

$$y_{ij}=\mu +T_{i}+S_{j}+\varepsilon_{ij}$$

where $S_{j}\in N\left(0,\sigma_{1}^{2}\right)$, *ε_ij _*∈ *N*(0, *σ*^2^). Here, *μ *is the mean expression value, *T_i _*is the fixed group effect (caused by the experimental conditions or treatments being evaluated), *S_j _*is the random sample effect (random effects from either individual biological samples or sample preparations), and *ε_ij _*is the within-groups errors. All random effects are assumed independent of each other and independent of the within-groups errors *ε_ij_*.

### Statistics

Statistical Significance was measured by a three-step method. First, we conducted the above linear mixed model to obtain the p value of the significance for the group effect. Then we calculated the FDR adjusted p value. Lastly, we calculated the FDR q value using the Storey-Tibshirani method [7]. We chose a significance screening filter (*q *< 0.01) to select genes of which we estimated significant differences in the health and breast cancer samples. The False Positive Rate (FPR), or expected proportion of false positive among the proteins with declared changes, is FPR = qvalue × number of the genes with declared changes.

### Support Vector Machine

The classification problem of breast cancer can be restricted to consideration of the two-class problem without loss of generality (breast cancer and normal). We developed a Support Vector Machine Recursive Feature Elimination(SVM-RFE) method [6] based on Cross-Validation (CV) (SVM-RFE-CV) to eliminate features for breast cancer from peripheral blood. And then we built a classifier based on the selected features and applied the classifier to predict breast cancer from peripheral blood in an independent testing set.

Consider the problem of separating the set of training patterns belonging to two separate classes (1, breast cancer; -1, normal), D = {(x_1_, y_1_),..., (x_1_, y_1_)}, x ∈ ℝ^n^, y∈ {-1, 1} with a hyperplane <*w, × *> + *b *= 0. The set of patterns is said to be optimally separated by the hyperplane if it is separated without error and the distance between the closest pattern to the hyperplane is maximal. Without loss of generality it is appropriate to consider a canonical hyperplane [8], where the parameters w, b are constrained by $\underset{\text{i}}{\min}|<w,{\text{x}}_{\text{i}}>+\text{b}|\;=1$. That is, the norm of the weight vector should be equal to the inverse of the distance, of the nearest point in the data set to the hyperplane. A separating hyperplane in canonical form must satisfy the following constraints: y_i_[<*w*, x_i _> +b] ≥ 1 - e_i_, i = 1, ..., l. Therefore, according to the structural risk minimization inductive principle, the training of an SVM is to minimize the guaranteed risk bound, $\underset{\text{w,b,e}}{\min}\text{ϕ}\text{(w,b,e)}=\frac{1}{2}{\text{W}}^{\text{T}}\text{W + }\frac{1}{2}\text{C }\sum_{\text{i}=1}^{1}{\text{e}}_{\text{i}}^{2}$, subject to the constraints y_i_[<*w*, x_i _> + b] ≥ 1 - e_i_, i = 1, ..., l.

The above optimization problem can be used in a linear recognition problem, but in this case, the classification problem is nonlinear. To solve the nonlinear classification problem, we can map first the training data to another feature space *F *via a nonlinear map φ: ℝ^n ^→ F and then perform the above computations in *F*. We used Gaussian radius basis function (RBF) kernels function for SVM.

### Recursive feature elimination(SVM-RFE) method based on cross-validation

SVM-RFE was introduced by Guyon et al. for selecting genes from microarray data analysis for cancer classification [6]. It includes four steps: 1) Train an SVM on the training set; 2) calculate ranking criteria based on the SVM weights; 3) Eliminate features with the smallest ranking criterion; and 4) Repeat the process. The feature elimination method is sensitive to small perturbations of the training set. The features it extracts from training set might not have good prediction performance in an independent testing set. Therefore, we adopted leave-one-out cross validation method to improve the stability and robustness of SVM-RFE. In addition, we chose |*W*| as ranking criteria instead of *W*^2 ^in the SVM-RFE-CV algorithm. The SVM-RFE [6] chose $C_{i}=W_{i}^{2}$ as ranking criteria and eliminates the feature with smallest ranking criterion. The original optimization equation in SVM actually depends on the absolute value of weight |*W*|. Substituting $\frac{1}{2}W^{2}$ for |*W*| can change the non-convex optimization to a quadratic programming optimization which is more easy to solve mathematically. But when we loop the feature elimination based on leave-one-out cross-validation, $\frac{1}{2}W^{2}$ loses its advantages over |*W*| on convexity of optimization. And |*W*| has bigger ranking criteria than $\frac{1}{2}W^{2}$, which makes optimization selection more accurate. Therefore, we chose |*W*| as ranking criteria in the SVM-RFE-CV algorithm.

The SVM Recursive Feature Elimination method based on Cross-Validation (SVM-RFE-CV) is described as follows:

k = ***K***; *#*Select All features

for (i in 1:n) #n is the sample size

{

  Build a SVM using the ith sample as testing set and others as training set;

  Calculate the feature weight Wi and the error rate Ei;

}

Sum up weights: W = sum(abs(Wi));

Sum up error rates: E = sum(Ei);

E0 = E;

while (E < = E0)

{

  E0 = E;

  rkw = rank(W); #rank the feature score

  k = k[which(rkw > 1)]; # remove features with lowest feature score

  for (i in 1:n)

  {

     Build a SVM using the ith sample as testing set and others as training set;

     Calculate the feature weight Wi and the error rate Ei;

  }

  Sum up weights: W = sum(abs(Wi));

  Sum up error rates: E = sum(Ei);

}

The error rate is calculated by 1 minus accuracy. All error rates for the n cross validations are summed up as determination of loop iterations.

### Enumeration method for validation

The enumeration method based on feed forward neural network was built to identify optimal biomarkers panel by us [9]. Similarly, we designed an enumeration method based on SVM to verify whether the biomarkers panel identified by the SVM-RFE-CV in the training set has still best prediction performance for the testing set.

We randomly chose M combinations of N (for example M = 100,000 when N = 15 for fifteen-marker panel) out of all the 42 genes differentially expressed in the training set and built M SVMs. In order to find the optimal classifier, we presented an enumeration method that measures the area under the curve (AUC) for Receiver Operating Characteristics (ROC). First, we trained the M SVMs in the training set with five-fold cross-validation. Then, we measured the AUC for each of the M combinations in the testing set. Lastly, the combinations were ranked by

$$rank_{C}=AUC\left(SVM_{C},T\right),$$

where AUC is the area under the ROC curve of SVM prediction, SVM is the trained classifier, C is one of M combinations of picking N out of the 42 genes, and T is the testing set.

### Cross-validation

k-fold cross-validation was used to increase the number of estimates and improve the accuracy of the prediction model by avoiding the over-fitting. In k-fold cross-validation, the original sample is randomly partitioned into k subsamples. Of the k subsamples, a single subsample is retained as the validation data for testing the model, and the remaining k-1 subsamples are used as training data. The cross-validation process is then repeated k times, with each of the k subsamples used exactly once as the validation data. The k results from the folds then can be averaged to produce a single estimation. The advantage of this method over repeated random sub-sampling is that all observations are used for both training and validation, and each observation is used for validation only once. If k equals the sample size, this is called leave-one-out cross-validation.

### Performance measurements

The following five measurements were involved in our evaluation: (1) Sensitivity (also called recall), the proportion of actual positive pairs which are correctly identified; (2) Specificity, the proportion of negative pairs which are correctly identified; (3) Precision, the probability of correct positive prediction; (4) Accuracy, the proportion of correctly predicted pairs; and (5) Area Under the Curve.

$$\begin{array}{ccc} Sensitivity & =\frac{TP}{TP+FN} & \\ Specificity & =\frac{TN}{TP+FP}\\ Precision & =\frac{TP}{TP+FP}\\ Accuracy= & \frac{TP+TN}{TP+TN+FP+FN} \end{array}$$

### Pathway analysis

We performed the pathway analysis using the databases: Integrated Pathway Analysis Database (IPAD) http://bioinfo.hsc.unt.edu/ipad/[10].

## Results

We downloaded from the Gene Expression Omnibus (accession number GSE16443) [3] the 130 samples with 67 breast cancer and 63 healthy women. After we randomly divided the 130 samples into two groups, group A as training set and group B as testing set (Table 1), we obtained 32 health samples and 33 cancer samples in the training set and 31 health samples and 34 cancer samples in the testing set. No data from the testing set were utilized in 1) identification of peripheral blood markers or 2) development of the SVM model.

**Figure 1:**
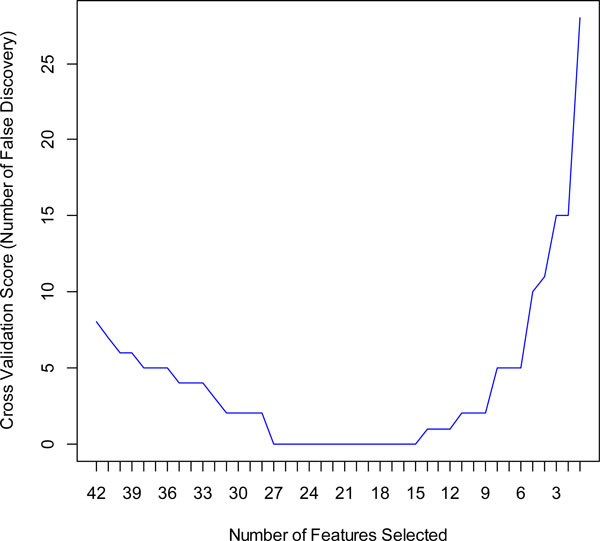
**Recursive feature elimination with automatic tuning of the number of features selected with cross-validation**.

We obtained 42 markers in the training set with qvalue < 0.01. An SVM model was built on all the 42 markers in the training set. We obtained a high performance (AUC = 0.98, precision = 97.0%, accuracy = 98.4%, sensitivity = 100.0%, specificity = 96.9%) for the training set but a low performance (AUC = 0.56, precision = 58.8%, accuracy = 56.9%, sensitivity = 58.8%, specificity = 54.8%) for testing set (Table 2). The result shows that using all markers as a predictor can achieve satisfactory prediction accuracy only for training set but not for the testing set. This is probably caused by overfitting which arises when 1) the number of features is large and the number of training patterns is comparatively small or 2) some regularities appear in the training data that do not appear in the test data. In order to avoid the overfitting and gain best prediction accuracy for the testing set, we built an SVM based on Recursive Feature Elimination and Cross Validation (SVM-RFE-CV) to extract optimal features. We show in Figure 1 the automatic tuning of number of features selected with the recursive feature elimination and the leave-one-out cross-validation. Training of the SVM-RFE-CV was performed using radius basis function (RBF) kernels function and leave-one-out cross-validation. Cross-validation scores were calculated to help evaluate the predictive performance of features selected by SVM-RFE-CV. The cross-validation scores is defined as number of false discovery which can be calculated by

**Table 2:** performance comparison of SVM, SVM-RFE, and SVM-RFE-CV

Measure	SVM		SVM-RFE		SVM-RFE-CV	
**#genes**	**42**		**18**		**15**	

	**Training set**	**Testing set**	**Training set**	**Testing set**	**Training set**	**Testing set**
Precision	97.0%	58.8%	100%	71.4%	100%	74.29%
Accuracy	98.4%	56.9%	100%	70.8%	100%	73.85%
Sensitivity	100.0%	58.8%	100%	73.5%	100%	76.47%
Specificity	96.9%	54.8%	100%	67.7%	100%	70.97%
AUC	0.98	0.56	1.0	0.75	1.0	0.80

cross-validationscore=ErrorRate*numberofsamplesize.

**Figure 2:**
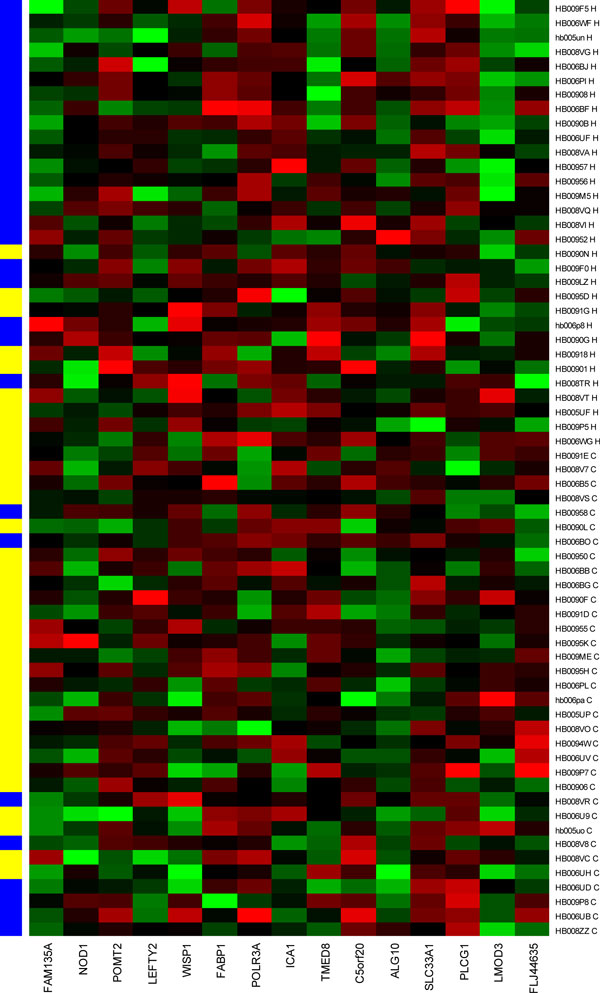
**15 biomarkers predicting the healthy and breast cancer samples in testing set**. X axis is the 15 biomarkers. Y-axis shows the 33 breast cancer and 32 healthy samples (H, healthy, blue; C, cancer, yellow).

The best cross-validation score with the least number of features occurs when the number of features is equal to 15 (Figure 1). The heatmap of the 15-marker panel for the testing set is shown in Figure 2. 26 out of 32 cancer samples and 22 out of 31 normal samples were correctly predicted.

Table 3 shows the direction and strength of expression changes for the 15 biomarkers. Some markers and their association with cancer already have been reported. For example, NOD1 is a cytosolic protein that senses meso-diaminopimelic acid-containing ligands derived from peptidoglycan and plays a role in host responses to invasive bacteria. Da et al. used Cell lines derived from the human breast cancer epithelial cell line (MCF-7) to characterize a pathway linking NOD1 to the growth of estrogen-sensitive tumors in a severe combined immune deficiency (SCID) mouse xenograft model. They found that the absence of NOD1 correlates with tumor growth, an increased sensitivity to estrogen-induced cell proliferation, and a failure to undergo NOD1-dependent apoptosis in the MCF-7 cells and conversely, overexpression of NOD1 in MCF-7 cells results in inhibition of estrogen-dependent tumor growth and reduction of estrogen-induced proliferative responses in vitro [11].

**Table 3:** gene expressions changes in 15-marker panel.

ProbeID	Genesymbol	GeneID	direction	qvalue
131318	FAM135A	57579	up	0.00775
134303	NOD1	10392	down	0.00995
146885	POMT2	29954	down	0.00459
154366	LEFTY2	7044	up	0.00928
155372	WISP1	8840	down	0.00197
162446	FABP1	2168	up	0.00735
167465	POLR3A	11128	down	0.00754
167529	ICA1	3382	down	0.00368
172360	TMED8	283578	up	0.00582
189547	C5orf20	140947	down	0.00941
206647	ALG10	84920	down	0.00050
210406	SLC33A1	9197	down	0.00483
211808	PLCG1	5335	down	0.00317
222602	LMOD3	56203	up	0.00508
230936	FLJ44635	392490	up	0.00773

Left-right determination factor 2 (LEFTY2) encodes a member of the TGF-beta family of proteins. The encoded protein is secreted and plays a role in left-right asymmetry determination of organ systems during development. The protein may also play a role in endometrial bleeding which is one of the most common manifestations of gynecologic diseases. Hernandez *et al. *found that promoters of LEFTY2 were differentially methylated in breast cancer samples relative to corresponding surrounding tissue [12].

Xie *et al. *observed overexpression of CTGF, WISP1, CYR61, and NOVH in primary breast tumors. They found significant associations between WISP1 mRNA levels versus stage, tumor size, lymph node, and HER-2/neu overexpression with statistical univariate analysis. Their results suggested that CTGF, WISP-1, and CYR61 might play a role in the progression of breast cancer and might serve as a valuable tool for monitoring tumor status of breast cancer patients [13]. Davies *et al. *analyzed the expression of the three WISP molecules at the mRNA and protein levels in a cohort of 122 human breast tumors and 32 normal breast tissues, and their correlations with patients' clinical outcomes. WISP1 transcripts were found in lower levels in node-positive tumors compared with node-negative tumors (P < .05); were lower in patients with a moderate (P = .01) and poor Nottingham Prognostic Index prognosis (P < .05) compared with good prognostic groups; were of significantly lower level in grade 3 differentiated tumors (P < .05) compared with grade 1; and were of lower levels in patients who developed metastasis and died from breast cancer-related causes (P < .05 in both comparisons). They concluded that WISP-1 seemed to act as a tumor suppressor, WISP-2 as a factor that stimulates aggressiveness; and WISP-3 has no definable beneficial or detrimental role [14].

FABP1 encodes the fatty acid-binding protein found in liver. Hammamieh *et al. *showed that blocking the expression of FABP1 resulted in remarkable effects on apoptosis and cell proliferation of prostate cancer cell lines [15] and FABP1 and intestine fatty acid binding proteins was up-regulated in breast cancer cell lines [16].

Sala et al. studied PLCG1 and its role in breast cancer metastasis and discovered this gene can promote cancer metastasis and subsequently blocking it stopped cancer from spreading. They showed that down-regulation of PLCgamma1 expression severely impaired activation of the small GTP-binding protein Rac and cell invasion in breast cancer cell lines and U87 in vitro. In addition, they found an increase of PLCgamma1 expression in metastasis compared with the primary tumor in 50% of 60 breast cancer patients' tissues analyzed. They suggested that PLCgamma1 inhibition had a therapeutic potential in the treatment of metastasis dissemination [17].

Arteaga et al. determined the relative content of PLC-gamma 1 in primary human mammary carcinomas and in nonmalignant mammary tissues. They detected considerably higher levels of PLC-gamma 1 protein in the majority of carcinomas and in one of two benign fibroadenomas compared to normal breast tissues by Western blot and immunohistochemistry. They also detected the presence of phosphotyrosine on PLC-gamma 1 in 18 of 21 carcinomas that contained high levels of PLC-gamma 1. They found that all carcinomas in which tyrosine phosphorylated PLC-gamma 1 was present also expressed detectable levels of the epidermal growth factor receptor or erbB-2, two tyrosine kinases known to phosphorylate this enzyme. They concluded that increased levels of receptor tyrosine kinases and a direct tyrosine phosphorylation substrate could be linked with a high percentage of mammary carcinomas and amplify two successive steps in a signal transduction pathway [18].

Pathway analysis shows the pathways linked with the fifteen-marker panel are signaling, hemostasis, hormone, and immune system (Additional file 1), which are consistent with previous findings [3].

**Figure 3:**
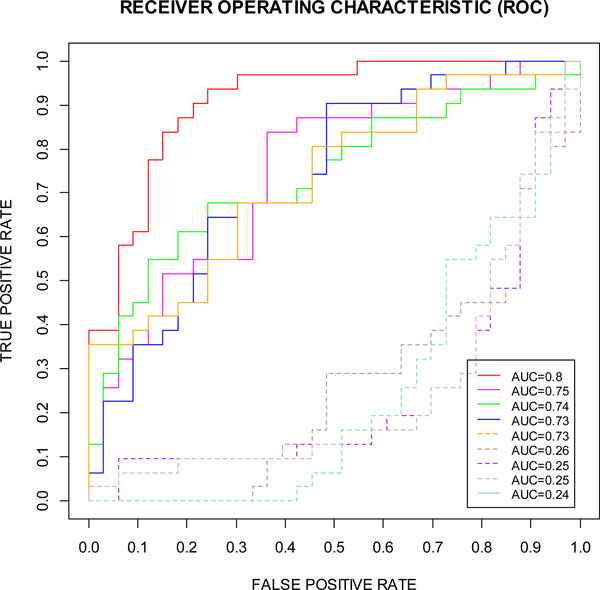
**Our 15-marker panel compared to 4 best randomly selected 15-marker panels (solid lines, out of 42 candidates) and 4 worst randomly selected 15-marker panels (dotted lines, out of 42 candidates)**. The 15-marker panel was compared with the best four 15-marker panels (solid lines) and the worst four 15-marker panels (dotted lines) which were randomly selected out of the 42 candidates.

The confusion matrix and common performance metrics for both the training data set and testing data set for the 15-marker panel is shown in Table 4. Although the final accuracy is 73.85% but can be considered as an improvement if compared to the original accuracy 56.9%. In addition, the AUC, a comprehensive measurement of sensitivity and specificity, is improved markedly from 0.56 to 0.8 (Figure 3 and Table 4).

**Table 4:** prediction result for the 15-marker panel

Training set			Testing set	
Predicted	Cancer	Normal	Cancer	Normal
Cancer	33	0	26	9
Normal	0	32	8	22
Precision	100%		74.29%	
Accuracy	100%		73.85%	
Sensitivity	100%		76.47%	
Specificity	100%		70.97%	

In order to validate our prediction method, we compared the ROCs for the 15-marker panel determined by the SVM-RFE-CV with the ROCs for 4 best randomly selected 15-marker panels and 4 worst randomly selected 15-marker panels from the 42 candidate biomarkers (Figure 3) using the Enumeration Method for Validation described in the method section. As shown in the Figure 3, the 15-marker panel determined by the SVM-RFE-CV (red solid line) has best sensitivity-specificity-tradeoff performance than others chosen randomly from 42 candidate biomarkers.

We also compared the classification and prediction performances of the three algorithms: SVM, SVM-RFE, and SVM-RFE-CV (Table 2). All the three algorithms can have high performance for the training. The performances of SVM-RFE and SVM-RFE-CV for the training set are the same. But there are differences in the number of genes and the performance for the testing set between the three algorithms. The SVM method has no function of feature elimination. The number of genes selected by SVM-RFE-CV is lower than that selected by SVM-RFE. In the testing set, the performance of SVM-RFE-CV is better than SVM-RFE.

We further evaluated our multi-marker panel prediction performance by comparing our results with prediction performance in previously published findings. Sharma *et al. *identified a panel of 37 genes that permitted early detection with the classification accuracy of 82% [4] and Aaroe *et al. *identified a set of 738 differentially expressed probes that achieved an estimated prediction accuracy of 79.5% with a sensitivity of 80.6% and a specificity of 78.3% [3]. Considering their methods were not applied to independent testing sets randomly separated from training set but used *k*-fold cross validation where the original sample was randomly partitioned into *k *subsamples and of the *k *subsamples, a single subsample was retained as the validation data for testing the model, and the remaining *k *- 1 subsamples were used as training data, our prediction performance actually outperformed them (Precision = 100%, Accuracy = 100%, Sensitivity = 100%, Specificity = 100%). We believe our approach is a significant success, considering that we only used fifteen gene markers in a panel to achieve the prediction performance (AUC = 0.8, Precision = 74.29%, Accuracy = 73.85%, Sensitivity = 76.47%, Specificity = 70.97%).

Lastly, we investigated the effect of qvalue's threshold on the results. We obtained 5 markers in the training set with qvalue < 0.001 and 1454 markers with qvalue < 0.1. For qvalue < 0.001, 3 markers were chosen With SVM-RFE-CV and produced the prediction performance of AUC = 0.5; for qvalue < 0.1, 16 markers were chosen With SVM-RFE-CV and produced the prediction performance of AUC = 0.78. Our results show that the qvalue < 0.01 has the best prediction performance with the SVM-RFECV.

## Conclusions

We developed an integrated computational approach that addressed a challenging multi-panel biomarker development problem in the early detection of breast cancer in peripheral blood. The approach combined recursive feature elimination of SVM with cross-validation. It automatically learned non-linear relationships between features and outcomes to generate the optimal predictive model with the least number of features, which achieved AUC = 0.80 performance with a sensitivity of 76.47% and a specificity of 70.97% in the testing data set of 34 women with breast cancer and 31 healthy women controls.

The SVM-RFE based on cross-validation is able to identify the optimal multi-markers panel with the least number of genes. It can filter irrelevant, tissue-specific genes from those related to malignancy. It also identifies gene expression patterns related to severity of disease. It is an effective method for finding markers implicated in cancers. In the future, we will follow up with biological experiments to validate these biomarkers we identified with our collaborators.

## Competing interests

The authors declare that they have no competing financial interests.

## Authors' contributions

YD and RD conceived the initial work and designed the method. FZ and YD downloaded the datasets, developed the classification method, and performed the statistical analyses. YD and HLK validated markers for early detection of breast cancer in peripheral blood. All authors are involved in the drafting and revisions of the manuscript.

## Supplementary Material

Additional file 1 **Pathway analysis for the fifteen-marker panel**. Click here for file

## References

[B1] Zhang F Chen JY Discovery of pathway biomarkers from coupled proteomics and systems biology methods BMC genomics 2010 11 Suppl 2 S12 10.1186/1471-2164-11-S2-S12 PMC2975409 21047379

[B2] Polyak K Breast cancer: origins and evolution J Clin Invest 2007 117 11 3155 3163 10.1172/JCI33295 17975657 PMC2045618

[B3] Aaroe J Lindahl T Dumeaux V Saebo S Tobin D Hagen N Skaane P Lonneborg A Sharma P Borresen-Dale AL Gene expression profiling of peripheral blood cells for early detection of breast cancer Breast cancer research: BCR 2010 12 1 R7 10.1186/bcr2472 20078854 PMC2880427

[B4] Sharma P Sahni NS Tibshirani R Skaane P Urdal P Berghagen H Jensen M Kristiansen L Moen C Zaka A Early detection of breast cancer based on gene-expression patterns in peripheral blood cells Breast cancer research: BCR 2005 7 5 R634 644 10.1186/bcr1203 16168108 PMC1242124

[B5] Li W How many genes are needed for early detection of breast cancer, based on gene expression patterns in peripheral blood cells? Breast cancer research: BCR 2005 7 5 E5 10.1186/bcr1295 16168099 PMC1242153

[B6] Guyon I Weston J Barnhill S Vapnik V Gene Selection for Cancer Classification using Support Vector Machines Mach Learn 2002 46 1-3 389 422

[B7] Storey JD Tibshirani R Statistical significance for genomewide studies Proc Natl Acad Sci USA 2003 100 16 9440 9445 10.1073/pnas.1530509100 12883005 PMC170937

[B8] Vapnik VN Statistical Learning Theory Springer, NY 1998 10.1109/72.788640 18252602

[B9] Fan Z A neural network approach to multi-biomarker panel development based on LC/MS/MS proteomics profiles: a case study in breast cancer 2009 1 6

[B10] Zhang F Drabier R IPAD: the Integrated Pathway Analysis Database for systematic enrichment analysis BMC Bioinformatics 2012 13 14 10.1186/1471-2105-13-S15-S7 PMC3439721 23046449

[B11] da Silva Correia J Miranda Y Austin-Brown N Hsu J Mathison J Xiang R Zhou H Li Q Han J Ulevitch RJ Nod1-dependent control of tumor growth Proceedings of the National Academy of Sciences of the United States of America 2006 103 6 1840 1845 10.1073/pnas.0509228103 16446438 PMC1413646

[B12] Hernandez-Vargas H Ouzounova M Le Calvez-Kelm F Lambert MP McKay-Chopin S Tavtigian SV Puisieux A Matar C Herceg Z Methylome analysis reveals Jak-STAT pathway deregulation in putative breast cancer stem cells Epigenetics: official journal of the DNA Methylation Society 2011 6 4 428 439 10.4161/epi.6.4.14515 21266853

[B13] Xie D Nakachi K Wang H Elashoff R Koeffler HP Elevated levels of connective tissue growth factor, WISP-1, and CYR61 in primary breast cancers associated with more advanced features Cancer research 2001 61 24 8917 8923 11751417

[B14] Davies SR Watkins G Mansel RE Jiang WG Differential expression and prognostic implications of the CCN family members WISP-1, WISP-2, and WISP-3 in human breast cancer Annals of surgical oncology 2007 14 6 1909 1918 10.1245/s10434-007-9376-x 17406949

[B15] De Santis ML Hammamieh R Das R Jett M Adipocyte-fatty acid binding protein induces apoptosis in DU145 prostate cancer cells Journal of experimental therapeutics & oncology 2004 4 2 91 100 15500004

[B16] Hammamieh R Chakraborty N Barmada M Das R Jett M Expression patterns of fatty acid binding proteins in breast cancer cells Journal of experimental therapeutics & oncology 2005 5 2 133 143 16471039

[B17] Sala G Dituri F Raimondi C Previdi S Maffucci T Mazzoletti M Rossi C Iezzi M Lattanzio R Piantelli M Phospholipase Cgamma1 is required for metastasis development and progression Cancer research 2008 68 24 10187 10196 10.1158/0008-5472.CAN-08-1181 19074886

[B18] Arteaga CL Johnson MD Todderud G Coffey RJ Carpenter G Page DL Elevated content of the tyrosine kinase substrate phospholipase C-gamma 1 in primary human breast carcinomas Proceedings of the National Academy of Sciences of the United States of America 1991 88 23 10435 10439 10.1073/pnas.88.23.10435 1683701 PMC52943

