# Supplementary material for: Recursive SVM biomarker selection for early detection of breast cancer in peripheral blood
Source: BMC Med Genomics. 2013 Jan 23;6(Suppl 1):S4. doi: 10.1186/1755-8794-6-S1-S4 (PMC3552693; doi:10.1186/1755-8794-6-S1-S4)
Supplement: Additional file 1 — Pathway analysis for the fifteen-marker panel. [file 1755-8794-6-S1-S4-S1.docx]

Additional file 1 pathway analysis for the fifteen-marker panel.

| PathwayID | PathwayName | Molecule |
| --- | --- | --- |
| 446193 | Biosynthesis of the N-glycan precursor (dolichol lipid-linked oligosaccharide, LLO) and transfer to a nascent protein | ALG10;ALG10 |
| 446203 | Asparagine N-linked glycosylation | ALG10;ALG10 |
| 597592 | Post-translational protein modification | ALG10;ALG10 |
| hsa00510 | N-Glycan biosynthesis | ALG10;ALG10 |
| 392499 | Metabolism of proteins | ALG10;ALG10 |
| h_pparaPathway | Mechanism of Gene Regulation by Peroxisome Proliferators via PPARa(alpha) | FABP1 |
| hsa03320 | PPAR signaling pathway | FABP1 |
| hsa04975 | Fat digestion and absorption | FABP1 |
| hsa04940 | Type I diabetes mellitus | ICA1 |
| 1433617 | Regulation of Signaling by NODAL | LEFTY2 |
| 1181150 | Signaling by NODAL | LEFTY2 |
| 114608 | Platelet degranulation | LEFTY2 |
| 76005 | Response to elevated platelet cytosolic Ca2+ | LEFTY2 |
| hsa04350 | TGF-beta signaling pathway | LEFTY2 |
| 445989 | TAK1 activates NFkB by phosphorylation and activation of IKKs complex | NOD1 |
| 450321 | JNK (c-Jun kinases) phosphorylation and activation mediated by activated human TAK1 | NOD1 |
| 168638 | NOD1/2 Signaling Pathway | NOD1 |
| 450302 | activated TAK1 mediates p38 MAPK activation | NOD1 |
| 446652 | Interleukin-1 signaling | NOD1 |
| 168643 | Nucleotide-binding domain, leucine rich repeat containing receptor (NLR) signaling pathways | NOD1 |
| 450294 | MAP kinase activation in TLR cascade | NOD1 |
| 1485579 | TRIF mediated TLR3 signaling | NOD1 |
| 166166 | MyD88-independent cascade initiated on plasma membrane | NOD1 |
| 168138 | Toll Like Receptor 9 (TLR9) Cascade | NOD1 |
| 168142 | Toll Like Receptor 10 (TLR10) Cascade | NOD1 |
| 168179 | Toll Like Receptor TLR1:TLR2 Cascade | NOD1 |
| 181438 | Toll Like Receptor 2 (TLR2) Cascade | NOD1 |
| 166058 | MyD88:Mal cascade initiated on plasma membrane | NOD1 |
| 168164 | Toll Like Receptor 3 (TLR3) Cascade | NOD1 |
| 168176 | Toll Like Receptor 5 (TLR5) Cascade | NOD1 |
| 168180 | TRAF6 Mediated Induction of proinflammatory cytokines | NOD1 |
| 168181 | Toll Like Receptor 7/8 (TLR7/8) Cascade | NOD1 |
| 168188 | Toll Like Receptor TLR6:TLR2 Cascade | NOD1 |
| 937061 | NFkB and MAP kinases activation mediated by TLR4 signaling repertoire | NOD1 |
| 975138 | TRAF6 mediated induction of NFkB and MAP kinases upon TLR7/8 or 9 activation | NOD1 |
| 975155 | MyD88 dependent cascade initiated on endosome | NOD1 |
| 975871 | MyD88 cascade initiated on plasma membrane | NOD1 |
| hsa05133 | Pertussis | NOD1 |
| hsa04621 | NOD-like receptor signaling pathway | NOD1 |
| hsa05131 | Shigellosis | NOD1 |
| 1251932 | PLCG1 events in ERBB2 signaling | PLCG1 |
| 167021 | PLC-gamma1 signalling | PLCG1 |
| 200085 | S1P1 pathway | PLCG1 |
| 200142 | EPHA forward signaling | PLCG1 |
| 200177 | VEGFR1 specific signals | PLCG1 |
| 200183 | EPO signaling pathway | PLCG1 |
| 200209 | N-cadherin signaling events | PLCG1 |
| 1236382 | Signaling by constitutively active EGFR | PLCG1 |
| 1489509 | DAG and IP3 signaling | PLCG1 |
| 200054 | S1P4 pathway | PLCG1 |
| 200071 | Regulation of CDC42 activity | PLCG1 |
| 200088 | Netrin-mediated signaling events | PLCG1 |
| 200101 | EGF receptor (ErbB1) signaling pathway | PLCG1 |
| 200147 | E-cadherin signaling in keratinocytes | PLCG1 |
| 200152 | Nephrin/Neph1 signaling in the kidney podocyte | PLCG1 |
| 200162 | PDGFR-alpha signaling pathway | PLCG1 |
| 200170 | Nongenotropic Androgen signaling | PLCG1 |
| 210990 | PECAM1 interactions | PLCG1 |
| 212718 | EGFR interacts with phospholipase C-gamma | PLCG1 |
| 418890 | Role of second messengers in netrin-1 signaling | PLCG1 |
| h_cxcr4Pathway | CXCR4 Signaling Pathway | PLCG1 |
| h_ghPathway | Growth Hormone Signaling Pathway | PLCG1 |
| h_ionPathway | Ion Channel and Phorbal Esters Signaling Pathway | PLCG1 |
| h_ngfPathway | Nerve growth factor pathway (NGF) | PLCG1 |
| h_TPOPathway | TPO Signaling Pathway | PLCG1 |
| h_trkaPathway | Trka Receptor Signaling Pathway | PLCG1 |
| h_vipPathway | Neuropeptides VIP and PACAP inhibit the apoptosis of activated T cells | PLCG1 |
| h_calcineurinPathway | Effects of calcineurin in Keratinocyte Differentiation | PLCG1 |
| h_cardiacegfPathway | Role of EGF Receptor Transactivation by GPCRs in Cardiac Hypertrophy | PLCG1 |
| h_Ccr5Pathway | Pertussis toxin-insensitive CCR5 Signaling in Macrophage | PLCG1 |
| h_egfPathway | EGF Signaling Pathway | PLCG1 |
| h_epoPathway | EPO Signaling Pathway | PLCG1 |
| h_erk5Pathway | Role of Erk5 in Neuronal Survival | PLCG1 |
| h_pdgfPathway | PDGF Signaling Pathway | PLCG1 |
| h_plcPathway | Phospholipase C Signaling Pathway | PLCG1 |
| h_ptdinsPathway | Phosphoinositides and their downstream targets. | PLCG1 |
| h_pyk2Pathway | Links between Pyk2 and Map Kinases | PLCG1 |
| h_vegfPathway | VEGF, Hypoxia, and Angiogenesis | PLCG1 |
| 200213 | Trk receptor signaling mediated by PI3K and PLC-gamma | PLCG1 |
| h_bcrPathway | BCR Signaling Pathway | PLCG1 |
| h_gpcrPathway | Signaling Pathway from G-Protein Families | PLCG1 |
| 373752 | Netrin-1 signaling | PLCG1 |
| 202433 | Generation of second messenger molecules | PLCG1 |
| h_fcer1Pathway | Fc Epsilon Receptor I Signaling in Mast Cells | PLCG1 |
| h_biopeptidesPathway | Bioactive Peptide Induced Signaling Pathway | PLCG1 |
| 200116 | Class I PI3K signaling events | PLCG1 |
| h_tcrPathway | T Cell Receptor Signaling Pathway | PLCG1 |
| 200075 | TCR signaling in na&#xef;ve CD8+ T cells | PLCG1 |
| 190347 | Phospholipase C-mediated cascade | PLCG1 |
| 200004 | Fc-epsilon receptor I signaling in mast cells | PLCG1 |
| 200188 | Signaling events mediated by VEGFR1 and VEGFR2 | PLCG1 |
| 200219 | FGF signaling pathway | PLCG1 |
| 200223 | Signaling events mediated by focal adhesion kinase | PLCG1 |
| 1169408 | ISG15 antiviral mechanism | PLCG1 |
| 1169410 | Antiviral mechanism by IFN-stimulated genes | PLCG1 |
| 200013 | LPA receptor mediated events | PLCG1 |
| 200027 | TCR signaling in na&#xef;ve CD4+ T cells | PLCG1 |
| 200041 | Signaling events mediated by Hepatocyte Growth Factor Receptor (c-Met) | PLCG1 |
| 200150 | Neurotrophic factor-mediated Trk receptor signaling | PLCG1 |
| 202403 | TCR signaling | PLCG1 |
| hsa04370 | VEGF signaling pathway | PLCG1 |
| hsa05110 | Vibrio cholerae infection | PLCG1 |
| hsa05214 | Glioma | PLCG1 |
| hsa05223 | Non-small cell lung cancer | PLCG1 |
| 109582 | Hemostasis | PLCG1;LEFTY2 |
| 1266738 | Developmental Biology | PLCG1;LEFTY2 |
| 1280215 | Cytokine Signaling in Immune system | PLCG1;NOD1 |
| hsa05120 | Epithelial cell signaling in Helicobacter pylori infection | PLCG1;NOD1 |
| 73780 | RNA Polymerase III Chain Elongation | POLR3A |
| 76046 | RNA Polymerase III Transcription Initiation | POLR3A |
| 76061 | RNA Polymerase III Transcription Initiation From Type 1 Promoter | POLR3A |
| 73980 | RNA Polymerase III Transcription Termination | POLR3A |
| 76066 | RNA Polymerase III Transcription Initiation From Type 2 Promoter | POLR3A |
| 76071 | RNA Polymerase III Transcription Initiation From Type 3 Promoter | POLR3A |
| hsa03020 | RNA polymerase | POLR3A |
| 74158 | RNA Polymerase III Transcription | POLR3A |
| 749476 | RNA Polymerase III Abortive And Retractive Initiation | POLR3A |
| hsa04623 | Cytosolic DNA-sensing pathway | POLR3A |
| hsa00514 | Other types of O-glycan biosynthesis | POMT2 |
| 425397 | Transport of vitamins, nucleosides, and related molecules | SLC33A1 |
| hsa00604 | Glycosphingolipid biosynthesis - ganglio series | SLC33A1 |
